# Microbial communities and soil chemical features associated with commercial production of the medicinal mushroom *Ganoderma lingzhi* in soil

**DOI:** 10.1038/s41598-019-52368-2

**Published:** 2019-11-01

**Authors:** Le-Qin Ke, Pu-Dong Li, Jian-Ping Xu, Qiu-Shuang Wang, Liang-Liang Wang, Hui-Ping Wen

**Affiliations:** 1 College of Ecology, Lishui University, Lishui City, Zhejiang Province 323000 P.R. China; 2 State Key Laboratory of Rice Biology, Institute of Biotechnology, Zhejiang University, Hangzhou, 310058 China; 3 Department of Biology, McMaster University, 1280 Main St. West, Hamilton Ontario, L8S 4K1 Canada; 4 Tea Research Institute, Guangdong Academy of Agricultural Sciences, Guangzhou, Guangdong 510006 P.R. China

**Keywords:** Microbiome, Fungi

## Abstract

Crop production, including mushroom farming, may cause significant changes to the underlying substrates which in turn, can influence crop quality and quantity during subsequent years. Here in this study, we analyzed the production of the medicinal mushroom *Ganoderma lingzhi* and the associated soil microbial communities and soil chemical features over 24 months from April 2015 to April 2017. This Basidiomycete mushroom, known as Lingzhi in China, is commonly found on dead trees and wood logs in temperate and subtropical forests. Its economic and medicinal importance have propelled the development of a diversity of cultivation methods. The dominant method uses wood logs as the main substrate, which after colonization by Lingzhi mycelia, are buried in the soil to induce fruiting. The soil microbial communities over the 24 months were analyzed using the Illumina HiSeq platform targeting a portion of the bacterial 16S rRNA gene and the fungal internal transcribed spacer 1 (ITS1). Overall, a significant reduction of Lingzhi yield was observed over our experimentation period. Interestingly, temporal changes in soil microbial compositions were detected during the 24 months, with the fungal community showing more changes than that of bacteria in terms of both species richness and the relative abundance of several dominant species after each fruiting. The soil chemical features also showed significant changes, with decreasing soil nitrogen and phosphorus concentrations and increasing soil pH and iron content after each fruiting. We discuss the implications of our results in sustainable Lingzhi production in soil.

## Introduction

Mushrooms have been cultivated and consumed by humans throughout the world for centuries. To date, there are more than 2000 known species of edible mushrooms and the consumption of most of these has been increasing in recent years. The reasons for the increased consumption include the increasing awareness of edible mushrooms’ health-promoting properties and their desirable culinary features^1^. Some edible mushrooms have shown potential as functional foods containing significant bioactive compounds of medicinal importance, such as polysaccharides, triterpenes, lectins, phenolics, and polyphenolics^2,3^.

The Chinese traditional medicine has a long history dating back thousands of years^4^. *Ganoderma lingzhi* (i.e. *Ganoderma lucidum* before 2012)^5^, called Lingzhi in Chinese, is a Basidiomycete fungus belonging to the family Polyporaceae and is among the best-known medicinal mushrooms^6^. This mushroom has been used for over 2,000 years as a Chinese traditional medicine and to some, it is considered a “herb of spiritual potency” that symbolizes health and longevity^7^. Previous studies have shown that bioactive compounds produced in this mushroom can inhibit adipocyte differentiation^8^ and reduce diabetes^9^, cancer^10^, and obesity-related disorders^11^. In addition, it has been shown to have antihyperlipidemic and antioxidant activities^12^.

In nature, this saprophytic mushroom grows well in temperate and subtropical regions on hardwood logs/chips, tree stumps and sawdust in warm and moist places. For commercial production, a variety of approaches have been used, including growing on wood logs and synthetic solid substrates for fruiting bodies, and in liquid culture for mycelia^13^. The dominant and most successful method was to grow the *G*. *lingzhi* mycelia in Linden wood logs, followed by burying the fully colonized logs into the soil. However, a major problem with this method is that there was significant reduction in yield after the first year and in subsequent planting. At present, the reason(s) for such a pattern is not known.

To help understand the potential mechanisms of the yield reduction in Lingzhi cultivated in soil, this study investigated the soil chemical features and soil microbial communities associated with Lingzhi cultivation. Specifically, we analyzed the chemical properties of soil samples in representative stages of Lingzhi growth and development over 24 months from April 2015 to April 2017. Similarly, the abundance, composition and taxonomic diversity of bacterial and fungal communities in these soil samples were investigated using culture-independent, high-throughput DNA sequencing method. The obtained data should help design appropriate management strategies for sustained production of Lingzhi.

## Materials and Methods

### Soil sampling

*Ganoderma lingzhi* cultivar Hunonglingzhi #1 was obtained from the Edible Mushroom Research Institute, Shanghai Academy of Agricultural Sciences (culture collection number 5768). The cultivar was grown in the soil in a greenhouse over 24 months, from April 2015 to April 2017, at the Senzhibao Biotechnology Company, Longquan, Zhejiang Province, China, following the protocol described below. Briefly, 30 cm long Linden wood logs of 15–22 cm in diameter were put into plastic bags (three logs per bag), sterilized with steam at 100 °C for 12 hours, and inoculated with actively growing mycelia of *G*. *lingzhi* cultivar Hunonglingzhi #1 at both ends of each log. After incubation at 22–25 °C for 45–60 days, the plastic bag covers were removed and the fully colonized logs were buried in soil in a greenhouse in early April 2015. In the soil, the logs were buried horizontally in rows, with ~10 cm of distance between rows. Within each row, the logs were separated by about 5 cm from each other. The logs were covered with 2 cm thick of soil. The logs were incubated in the greenhouse at 26–28 °C, with a relative humidity maintained at 80–95% until fruiting body formation in September and spore production in October. Over the ensuring 24 months, Lingzhi fruited once in October 2015 and the second time a year later, in October 2016. During those two fruiting periods, basidiospores were harvested by spore-trapping using specialized plastic bags fitted around individual fruiting bodies and their weight recorded. At the end of the basidiospore production seasons, in October 2015 and October 2016, all the fruiting bodies (with the spores already harvested) were respectively harvested and weighed. Soil samples were taken at four time points from the greenhouse and named as RS1, RS2, RS3 and RS4. At each time point, we obtained three repeats. The three RS1 soil samples were collected just before the burying of Lingzhi-colonized logs in the soil. The RS2 samples were collected in October 2015 when the first batch of Lingzhi spores and fruiting body were harvested. The three RS3 samples were taken a year later after RS2, in October 2016, during the second Lingzhi harvesting. The last three soil samples were taken in April 2017. For each of the 12 soil samples, we obtained ~100 grams of soil from 5–10 cm beneath the surface in-between where the rows of buried logs were placed with a sterilized stainless-steel shovel. The soil was thoroughly mixed with about half (i.e. ~50 g) put into an 50 ml sterilized tube and stored in a −80 °C freezer for extraction of soil DNA. The remaining 50 g were used for analyzing soil physical and chemical properties.

### Soil chemical analysis

The soil samples for chemical analysis were homogenized thoroughly by milling. The homogenized soil samples were all air-dried for 48 h and then passed through a 2-mm sieve to remove leaves, plant roots, and gravels. Soil pH was measured by pH meter HACH-HQ30d, following the protocol described below. The soil sample (10 g) was placed into a 100 mL beaker and mixed with 25 mL sterilized distilled water. After shaking for 5 min, the mixture was left for 20 min to equilibrate. The pH was then measured using a calibrated pH meter. The total available nitrogen in the soil samples was measured using the Kjeldahl method (NY/T 1121.24–2012)^14^. Total phosphorous was determined by HClO_4_-HF digestion followed by colorimetric analysis (NY/T 1121.7–2014)^15,16^. The available iron content in soil was measured by the diethylenetriaminepentaacetic acid (DTPA) based extraction method (HJ 804–2016)^17,18^.

### Genomic DNA extraction

For each of the 12 soil samples (four time periods x three replicates at each time period), 1 g of soil sample was used to extract total genomic DNA using the cetyltrimethylammonium bromide (CTAB) method^19^. The extracted soil DNA was dissolved in 100 μL TE buffer. Successful isolation of soil genomic DNA was visually confirmed using 1% agarose gel electrophoresis. DNA quality and quantity were measured using the NanoDrop 2000 Spectrophotometer (ThermoFisher Scientific). The obtained DNA samples were diluted to 1 ng/μL using sterile water and stored at −80 °C before use.

### Library preparation and sequencing of bacterial and fungal amplicons

To determine the diversity of bacterial and fungal communities in the soil samples, we used Illumina sequencing of bacterial 16S rRNA genes and fungal internal transcribed spacer (ITS) regions. The hypervariable V4 regions of the bacterial 16S-rRNA gene were amplified by universal primers 515 F and 806 R using the PCR conditions previously described by Caporaso *et al*.^20^. For analyses of fungal communities, the ITS1 and ITS2 primers to amplify the ITS-1 region of the fungal rRNA operons following the same PCR conditions as described in Monard *et al*.^21^. The sequences of the primers used to target 16S and ITS regions are given in Supplementary Table S1.

All PCR reactions were carried out with Phusion® High-Fidelity PCR Master Mix (New England Biolabs). PCR amplification products were purified using the QIAquick PCR Purification Kit (QIAGEN, Germany) and then quantified using a Qubit® 2.0 Fluorometer (Fisher Thermo Scientific). Sequencing libraries were generated using the TruSeq® DNA PCR-Free Sample Preparation Kit (Illumina, USA) following manufacturer’s recommendations. The library quality was assessed using the Qubit® 2.0 Fluorometer (Thermo Scientific) and Agilent Bioanalyzer 2100 system. The libraries were then sequenced using the Illumina HiSeq 2500 platform.

### Processing of Illumina data

Paired-end reads were assigned to each sample by their unique barcodes and truncated by cutting off the barcode and primer sequences. After initial trimming, paired-end reads were merged using FLASH (V1.2.7, http://ccb.jhu.edu/software/FLASH/)^20^. Low quality reads were then discarded using QIIME. Chimeras were detected with the gold reference database using the UCHIME algorithm and deleted^22^. Then, the UPARSE software (Uparse v7.0.1001, http://drive5.com/uparse/) was used to pick operational taxonomic units (OTUs)^23^. Sequences with ≥97% similarity were assigned to the same OTUs. After picking OTUs, one representative sequence from each OTU was selected, and using the Ribosomal Database Project (RDP) classifier, taxonomic data were assigned to each representative sequence of the OTU^24^.

### Diversity and statistical analyses

Except for rarefaction curves, OTU abundances were all normalized to the sample with the least sequence reads. Subsequent analysis of alpha diversity and beta diversity were all performed based on the normalized data. The alpha diversity indices such as the Chao1 richness, ACE and Shannon diversity index, were calculated in QIIME (Version 1.7.0) and displayed with R software (Version 2.15.3). These indices were used to compare bacterial and fungal alpha diversities among soil samples. Beta diversity analysis was used to evaluate differences among soil samples using QIIME (Version 1.7.0). The statistical significance of the differences in soil chemical properties, bacterial alpha diversity at various taxonomic levels was calculated using one-way-analysis of variance (ANOVA) followed by Duncan’s-test using the SPSS software (Version 18.0). Cluster analysis was preceded by principal component analysis (PCA), which was applied to reduce the dimension of the original variables using the FactoMineR package and ggplot2 package in R software (Version 2.15.3). Heatmap figures were generated using custom R scripts. Analysis of variance and Spearman’s rank correlations were performed using SPSS Statistics 18 (IBM, Armonk, New York, USA).

## Results and Discussion

After planting the *Ganoderma* mycelia-colonized logs in the greenhouse soil in April 2015, we quantified both the basidiospore production and the fruiting body weight during two fruiting seasons, in October 2015 and October 2016. The summary results are presented in Table 1. For both spore and fruiting body productions, the yields in the first fruiting season (in October 2015) were significantly higher than those in the second fruiting season (in October 2016) (p values < 0.001 in both cases). Because of the significant reduction in yields, farmers typically abandon or convert the Lingzhi-planted soils into other crops after two years of Lingzhi cultivation. In our study, the Lingzhi-colonized logs were removed in December 2016, after the fruiting season was finished in October 2016. To further quantify the potential effects of prior Lingzhi cultivation on subsequent Lingzhi production, Lingzhi-colonized logs were replanted in April 2017, right after our RS4 samples were taken. As shown in Table 1, the basidiospore and fruiting body yields for the new crops in October 2017 and October 2018 were significantly lower than the first sets of logs in their first and second fruiting seasons respectively.

**Table 1:** Lingzhi fruiting body and spore productions over three years (2015–2018) after planted in soil (unit: kg/100 m^2^).

Planting Cycle	Time of harvest	Product	Repeat #1	Repeat #2	Repeat #3	Mean ± standard deviation*
Cycle #1: logs buried in April 2015	October, 2015	Fruiting body	30.87	29.81	30.26	30.31 (±0.53)^a^
		Spore	43.13	45.48	44.75	44.45 (±1.20)^b^
	October, 2016	Fruiting body	14.58	15.95	15.29	15.27 (±0.69)^c^
		Spore	24.88	23.19	22.79	23.62 (±1.11)^d^
Cycle #2: logs buried in April 2017	October, 2017	Fruiting body	18.58	15.78	17.67	17.34 (±1.43)^c^
		Spore	20.64	14.76	17.56	17.65 (±2.94)^c^
	October, 2018	Fruiting body	7.68	8.53	10.51	8.91 (±1.45)^e^
		Spore	7.15	8.92	9.66	8.58 (±1.29)^e^

^*^The means with different superscripts are significantly different from each other at P < 0.05 according to Duncan’s multiple range tests.

### Soil chemical analysis

The results of soil chemical properties including pH, total nitrogen, available phosphorous, and iron contents were given in Table 2. The results showed overall gradual decreases in soil pH, total nitrogen and phosphorous contents with the increasing Lingzhi cultivation over the 24 months. The reductions in soil nitrogen and phosphorus are expected given that Lingzhi requires both elements to grow and reproduce. Interestingly, we observed a significant (P < 0.05) increase in biologically available iron content over the 24 months. A previous study has shown that trace elements like Zn and Fe are generally conducive to the growth and efficacy of edible mushrooms^25^. Each trace element has its unique physiological role in mushroom growth and microbial community structure^26^. In turn, the microbial community could also impact the concentration of available elements. For example, although the quantity of total Fe is generally high in soils, the level of available fraction is generally low in most natural environments. Microbes including Lingzhi may release enzymes and siderophores which can increase Fe availability by binding and solubilizing the Fe present within minerals^27^. Further analyses are needed in order to identify whether the observed differences were due to log planting, Lingzhi growth or a combination of both. Indeed, the influences of both the log and Lingzhi on the soil chemical properties were likely due to the organic and inorganic materials that they brought to the soil. In addition, if the changes were due to Lingzhi growth, it would be interesting to investigate whether the changes were the direct effects by Lingzhi growth or indirect effects by Lingzhi’s influence on other microbes.

**Table 2:** Chemical properties of soil samples. All data points represent “Mean + Standard deviation”.

Samples	pH	Total nitrogen g/kg	Available phosphorus mg/kg	Available Fe mg/kg
RS1	5.8 ± 0.4	2.38 ± 0.15	134.1 ± 2.3	144.1 ± 3.4
RS2	5.3 ± 0.3	2.31 ± 0.16	98.3 ± 1.3	173.3 ± 6.3
RS3	4.4 ± 0.4	2.07 ± 0.45	81.0 ± 2.1	221.4 ± 9.2
RS4	4.3 ± 0.1	1.88 ± 0.32	53.1 ± 1.1	264.7 ± 4.1

### General analyses of the sequencing data

In this study, the soil microbial communities at four time points associated with Lingzhi fruiting and sporulation from April 2015 to April 2017 were analyzed through high-throughput sequencing. We used primers and protocols described in previous studies for the bacterial 16S rRNA and the fungal ITS1 sequences for microbial metabarcoding^20,21,28,29^. A total of 733,164, and 707,856 raw reads were obtained for the 16S rRNA and the ITS1 sequences respectively from the 12 samples that included three biological repeats of each of the four sampling time points, RS1, RS2, RS3 and RS4 (Supplementary Table S2 and Table S3). After quality control that included filtering out short and low-quality reads, singletons, replicates, and chimera, an average of 65,327, 64,736, 50,609 and 51,099 clean reads for the 16S rRNA, and 43,888, 39,271, 48,722, 49,356 clean reads for the ITS1 were obtained for the samples RS1, RS2, RS3 and RS4, respectively. The average length (bp) of the sequences was 253 and 248 for 16S and ITS1, respectively. An average number of 4,763 OTUs, ranging from 4,040 to 5,399, were obtained for bacteria per sample (Table S1). Similarly, an average number of 1,363 OTUs, ranging from 1,242 to 1,556, for ITS1 (Table S2) were obtained from the four samples for fungi. The high read counts and large numbers of bacterial and fungal OTUs within individual samples allowed us to investigate the potential microbial changes in the soil rhizosphere associated with Lingzhi fruiting and spore production, as described below.

### Bacterial community dynamics

**Figure 1:**
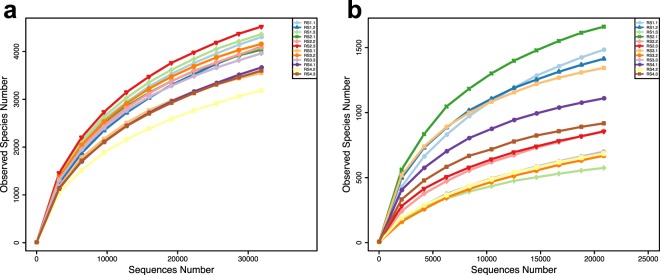
Rarefaction analysis indicating bacterial (**a**) and fungal (**b**) species richness in four soil samples RS1, RS2, RS3, and RS4.

The mean alpha diversity parameters (Chao1 richness, ACE diversity index and Shannon diversity index) of bacterial communities from soil samples at the four different time points are presented in Table 3. Out of these parameters, the rarefaction analysis of the OTUs, shown in Fig. 1, indicated that bacterial diversity and richness were higher at the beginning (RS1) and after the first year of the Lingzhi production (RS2). In contrast, the Shannon diversity index, the Chao1 index, and the total number of bacterial OTUs were the lowest in the soil RS4.

**Table 3:** The alpha diversity indices of bacteria and fungi in different soil samples. All data points represent “Mean + Standard deviation”.

Sample name	Observed species	Shannon	Chao1	ACE
**Alpha diversity indices for bacteria communities**				
RS1	4255 ± 137	10.26 ± 0.22	5677 ± 189	5910.3 ± 232
RS2	4215 ± 265	10.1 ± 0.20	5232.7 ± 473	5463.7 ± 404
RS3	3889.6 ± 309	10 ± 0.25	4743 ± 586	4947 ± 484
RS4	3483.6 ± 260	9.56 ± 0.23	4596.5 ± 558	5055.4 ± 527
**Alpha diversity indices for fungal communities**				
RS1	1157.6 ± 505	5.55 ± 2.17	1491.85 ± 642	1551.6 ± 700
RS2	1124.6 ± 464	5.36 ± 2.36	1486.4 ± 368	1530.2 ± 436
RS3	904.3 ± 381	5.10 ± 2.73	1206.7 ± 281	1312.2 ± 251
RS4	904.3 ± 212	4.66 ± 1.24	1133.1 ± 202	1193.4 ± 173

There is no previous report on the effects of Lingzhi growth on soil bacterial communities. However, it has been reported that the mushroom cropping could significantly affect the bacterial and fungal community structures of the casing soil during the production of the button mushroom *Agaricus bisporus*^30^. Furthermore, it has been suggested that soil physical chemical features including soil fertility could be impacted by mushroom cultivation. Our results are consistent with those suggestions. Whether such changes were due to the direct effects of Lingzhi, indirectly through changes of other microbes, or both remains to be examined. Indeed, it’s also possible that the buried logs or the log-Lingzhi combination were the drivers for the observed changes. However, since our soil samples were obtained away from the logs and without direct contacts with Lingzhi fruiting bodies, the changes we observed in the soil microbial communities were most likely due to diffusible substances released by either the logs or the Lingzhi or both.

**Figure 2:**
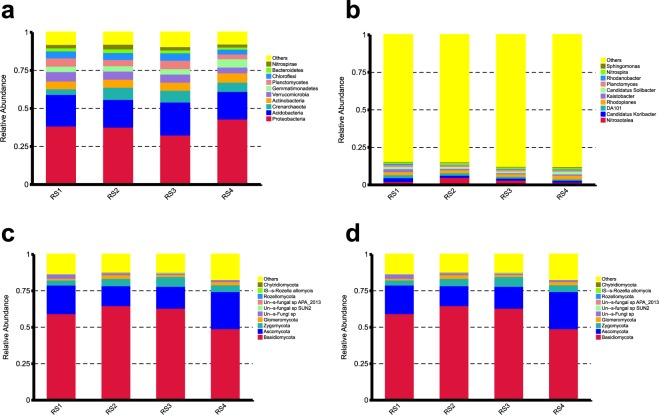
Classification of soil bacterial and fungal OTUs at the phylum and species levels. Graphs (**a**) and (**b**) show the relative abundance of dominant bacterial phyla and genera, respectively, inferred based on 16S rRNA gene sequences. Graphs (**c**,**d**) show the relative abundance of dominant fungal phyla and genera, respectively, inferred based on ITS sequences.

The filtered 16S rRNA sequence reads in each metabarcoding data set were taxonomically classified and presented in Supplementary Table 4 (Table S4). At the highest taxonomic level, 93.6% of the 16S rRNA reads belonged to Bacteria, with the remaining 6.4% belonging to Archaea. The taxonomic distribution at the phylum level is shown in Fig. 2a. *Proteobacteria* proved to be the most abundant phylum in all the four samples, accounting for 44.7% of the total reads in the sample RS4 and 38.4%, 37.7% and 33.4% of the total reads in RS1, RS2 and RS3, respectively. The high abundance of *Proteobacteria* in soil samples is not surprising and has been reported in many previous studies^31,32^. *Acidobacteria* was the second most abundant phylum in all our samples with an average relative abundance of 19.48%. In a previous study, the phylum *Acidobacteria* was shown as the most abundant (up to 73%) bacterial phylum in diverse environments^31^. Other common bacterial phyla included *Actinobacteria* (5.10–6.43%, averaging at 5.60%), *Verrucomicrobia* (3.40–6.35%, averaging at 5.15%), *Planctomycetes* (2.82–5.72%, averaging at 4.52%) and *Gemmatimonadetes* (3.52–5.60%, averaging at 4.08%). Most of these phyla are commonly found in soil samples. At the Class level, a number of bacterial classes were dominant, including *Alpha-proteobacteria*, *Acidobacteria*, *Delta-proteobacteria*, *Beta-proteobacteria* and *Acidobacteria-6*. Based on the average relative abundance of the most common taxonomic Orders, we found that some differences among the groups. For example, *Acidobacteriales* was the most abundant Order in samples RS1 and RS3 (8.25% and 7.92%, respectively), *Cenarchaeales* (7.51%) was the most abundant Order in RS2, while *Rhodospirillales* was the most abundant in sample RS4 (11.6%).

**Figure 3:**
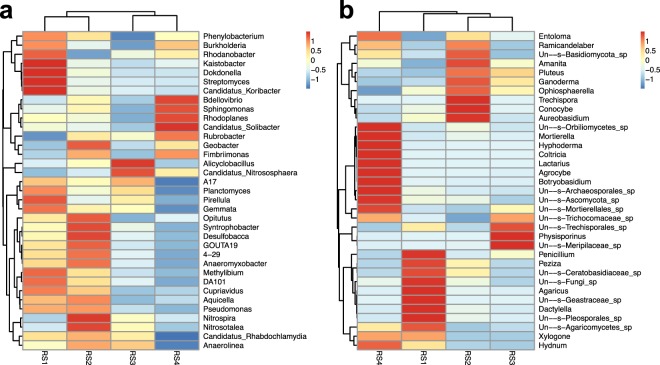
Heat-map of key bacterial (**a**) and fungal (**b**) taxa at the genus level.

The most common genera in different soil samples are shown in Fig. 2b and a heat-map of key bacterial taxa at the genus level is shown in Fig. 3a. The differences in microbial diversity at the genus level in the four soil samples varied broadly. For instance, *Candidatus Koribacter* was the most abundant genus (accounting for 2.5%) in RS1, whereas *Nitrosotalea* was the most abundant genus in other soil samples, but with slightly variable compositions: 5.12% in RS2, 3.35% in RS3, and 1.96% in RS4. *Candidatus Koribacter*, *Nitrosotalea*, *Kaistobacter*, *Rhodoplanes*, *Candidatus Solibacter*, *DA101* and *Planctomyces* were the most abundant genera in RS1, before the cultivation of Lingzhi. However, after two-years consecutive cultivations (RS4) of Lingzhi on the same soil, the abundance of *Candidatus Koribacter*, *DA101*, *Nitrosotalea*, *Planctomyces* and *Kaistobacter* significantly (P < 0.05) reduced. Unclassified taxa at the genus level were the highest in RS4 sample (82.2%) followed by RS3 (81.3%), RS2 (77.1%) and RS1 (76.7%). We would like to note that though the overall soil microbial composition varied among the four soil samples, the main genera remained the same over the 24 months.

### Fungal community dynamics

The mean alpha diversity parameters (Chao1 richness, ACE diversity index and Shannon diversity index) of the fungal communities from different soil samples are presented in Table 3. There was an overall decrease in fungal community Shannon and Chao1 indices after each consecutive year of growing Lingzhi. These findings were also confirmed from the rarefaction curves (Fig. 1b). These results indicate that Lingzhi cultivation using the buried colonized logs impacted the fungal community in the soil. In order to determine the taxonomic composition of fungal communities and what specific taxa had changed, all filtered reads were classified from phylum to genus levels using QIIME with default settings. The taxonomic classification of fungal communities at the phylum and genus levels is shown in Fig. 2(c,d). The total number of sequence reads in each metagenomic data set was taxonomically classified and presented in Supplementary Table 5 (Table S5).

The most abundant fungal phylum detected across all samples was *Basidiomycota* with an average abundance of 62.8% of all fungal sequences. However, some variations in the relative abundance of Basidiomycota were observed among the soil samples, with RS4 showing the lowest abundance of 53.4%. *Ascomycota* was the second dominant phylum with an average abundance of 17.6% in all samples followed by *Zygomycota* with an average abundance of 4.38%. The highest abundance of *Zygomycota* was observed in sample RS4 (23.7%). The relatively high abundances of Basidiomycota and Ascomycota in soil are in agreements with findings in many previous studies. For instance, McGuire *et al*.^33^ and Schmidt *et al*.^34^ reported that these two phyla accounted for more than 60% of all sequences in soil samples. At the genus level, an interesting pattern of taxa abundance among soil samples emerged. Specifically, *Ganoderma* was the most abundant genus in RS2, accounting for about 50% of the reads. However, in other three samples, it accounted for 9.3% of the reads in RS1, 29.4% in RS3 and only 3.8% in RS4. This abundance pattern is consistent with what we expected during the production cycle of Lingzhi in the soil. For instance, its abundance was low before the plantation of Lingzhi (RS1, at 9.3%) and six months after the October 2016 fruiting season ended and the logs removed (RS4, at 3.8%). In between, with the abundance of Lingzhi mycelia in the logs and the basidiospores from the fruiting bodies that escaped the trapping, the ITS1 counts of *Ganoderma* reached 50% and 29.4% respectively in RS2 and RS3 samples respectively. The lower counts of *Ganoderma* in RS3 than RS2 could be due to the low yield in the October 2016 harvest.

Interestingly, though its abundance was very low in the earlier samples ( < 1% in RS1, RS2 and RS3), *Hyphoderma* was the most abundant genus in RS4 accounting for 29.6% of the total reads. The genus *Agaricus* showed a continuous decrease through the three years. Specifically, the abundance of *Agaricus* decreased from 18.5%, before the growth of Lingzhi (RS1), to 0.56%, 0.09% and 0.04% after first (RS2), second (RS3) and third (RS4) year of the growth of Lingzhi, respectively. The soil sample RS4 was distinct from rest of the samples due to the dominance of few genera that were rare in other samples. Those genera included *Hypoderma*, *Agrocybe*, *Mortierella*, *Coltricia*, *Lactarius* and *Botryobasidium* (Fig. 3b).

**Figure 4:**
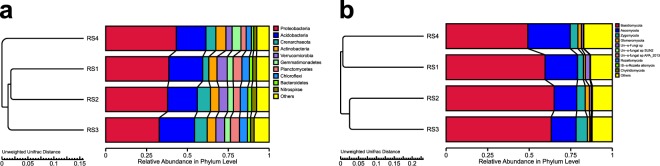
UPGMA clustering of soil bacterial (**a**) and fungal (**b**) communities, at the phylum level, associated with different soil samples obtained from the Lingzhi fields before and after the consecutive Lingzhi cultivation based on un-weighted Unifrac distance.

The pattern shown at the genus level also showed up at the species level. *Ganoderma lingzhi* was the highest after the first year of Lingzhi production, accounting for 50.3% of the total reads. Its abundance decreased over the following two years, to 29.4% and 3.8% respectively in RS3 and RS4. Similarly, *Hyphoderma nudicephalum* appeared as the most dominant species in RS4 accounting for 29.5% of the total reads. The abundance of *Agaricus* sp. F2272 decreased from 18.5%, before the growth of Lingzhi (RS1), to 0.56%, 0.09% and 0.04% at the first (RS2), second (RS3) and third (RS4) sampling points, respectively. Taken together, the results suggested that *Agaricus* species in soil was likely negatively affected by Lingzhi production. However, we can’t exclude the possibility that such changes were part of naturally fluctuations in soil fungal flora around our experimentation site. Soil samples from comparable sites but with non-Lingzhi production are needed in order to investigate this possibility. Result of cluster analysis among the samples using the UPGMA method based on the Unifrac distance is shown in Fig. 4.

### Relationships between alpha-diversity and environmental variables

The relationship between alpha-diversity and soil chemical properties is given in Table 4. In the case of bacteria, the number of OTUs, Shannon index and the value of Chao1 showed significant positive correlation with soil total nitrogen, phosphorous and iron contents. The values of Chao1 also showed a significant positive correlation with soil pH. However, no significant correlation was found between soil pH and the number of OTUs or the Shannon index. The results suggest that the growth and production of Lingzhi have likely depleted the levels of total soil nitrogen and phosphorous which subsequently affected the type of bacteria that can grow in such environments. Decreases in nitrogen and other nutrients have been found to negatively affect microbial alpha-diversity in soil^35^. In this study, a decrease in available nitrogen was found to be correlated with a decrease in the relative abundance of *Nitrospirae* from 3% (RS2) to 1.88% (RS4). *Nitrospirae* are nitrite-oxidizing chemolithoautotrophic bacteria that plays a key role in nitrification and the nitrogen cycle in natural environments^36^. However, the statistical and biological significance of the 1.2% reduction in the relative abundance of *Nitrospirae* in our experimental plots to Lingzhi production requires further examination. For example, future experiments could include analyzing the relative concentrations of nitrogen in different oxidative states in the soils with and without Lingzhi cultivation and targeted treatments by manipulation the soil nitrogen contents using fertilizers.

**Table 4:** Pearson’s correlation coefficients between soil characteristics and bacterial and fungal alpha diversity^a^.

		pH	Total nitrogen	Phosphorous	Iron
Bacteria	OTU	0.80	0.91*	0.97**	−0.94*
	Shannon	0.69	0.89*	0.88*	−0.90*
	Chao1	0.98**	0.89*	0.94*	−0.93*
Fungi	OTU	0.97**	0.81	0.9*	−0.86
	Shannon	0.82	0.87**	0.93*	−0.97**
	Chao1	0.91*	0.95*	0.78	−0.92*

^a^The result from average value of parameters compared for the correlation.

*Correlation is significant at the 0.05 level.

**Correlation is significant at the 0.01 level.

Our analyses showed that the number of fungal OTUs, Shannon index and Chao1 all had a positive correlation with measured soil nutrients, except with iron where a negative correlation was observed. For example, we found significant positive correlations between the number of OTUs and soil pH and phosphorus content; between the Shannon index and soil nitrogen and phosphorous contents; and between Chao1 index and soil pH and nitrogen content. Similar to what was observed for the bacterial community, the soil fungal community after Lingzhi production showed a negative correlation with soil iron content. Together, these results indicate that the structures of bacterial and fungal communities are closely related to soil chemical properties in Lingzhi production fields and can be shaped by soil pH, nitrogen and phosphorus contents (P = 0.05 or 0.01). For instance, the growth of arbuscular mycorrhizal (AM) fungi is negatively regulated by soil phosphorus and positively influenced by soil nitrogen content^37^.

Soil pH has shown to be among the most important chemical properties that influence bacterial and fungal composition and richness in soil^38^. In our study, changes in soil pH were correlated with changes in bacterial and fungal diversities. An exception was the lack of significant correlation between soil pH and bacterial diversity and richness as indicated by the Shannon index and OTUs. A possible explanation of this observation may be the negative effect of pH decrease on bacterial diversity. Most bacteria prefer to grow at neutral pH while most fungi prefer to grow at acidic pH environments. In the soil, certain bacteria and certain fungi play important roles in regulating pH. In our soil samples, *Acidobacteria* was among the most common genera and bacteria in this genus play a key role in regulating the pH value of soil^39^. Jones *et al*.^40^ reported that increasing abundance of *Acidobacteria* decreases soil pH. Our results are consistent with the major effect of this group of bacteria in regulating soil pH and how the pH decrease could negatively impact bacterial diversity.

## Conclusions and Perspectives

This study investigated the potential effects of Lingzhi cultivation using the buried log method on soil microbial communities (bacteria and fungi) and soil chemical features. Our results showed that this method of Lingzhi cultivation decreased the total soil nitrogen and phosphorous contents while increased the available iron. Interestingly, soil pH also decreased significantly, likely due to the increasing prevalence of *Acidobacteria* and changes in other microbial flora accumulated during the Lingzhi growth cycle. Significant changes in both the bacterial and fungal microbial communities were observed through the 24 months during our experiments. At present, the mechanisms for such changes are not known. In addition, though the loss of Lingzhi productivity (in both basidiospore and fruiting body weight) has been similarly reported using the buried-log method in other geographic regions, it is currently unknown whether there are changes similar to those observed here in the soil chemical and microbiological features in those areas. Thus, additional research is needed to confirm the broad significance of our observations here. Furthermore, we note that some of those changes were relatively small and could be caused by experimental procedures and artifacts such as PCR amplification bias. However, it’s tempting to speculate that the growth and production of Lingzhi caused the changes in soil biotic and abiotic features and in turn, such changes may have negatively influenced the growth and production of Lingzhi in subsequent years. Our results also suggest potential approaches to mitigate the decrease of Lingzhi production over successive cultivations. For example, nitrogen and phosphate fertilizers could be added to increase their availability and calcium carbonate could be added to increase pH. Controlled experiments are needed in order to test the specific effects of those changes in the soil on Lingzhi growth and production. The data generated here provide the bases from which to design specific experiments and test these hypotheses.

## Supplementary information

Supplementary table

## Data Availability

The DNA reads have been deposited at NCBI under the bioproject accession No. PRJNA562418.
