# Supplementary material for: Microbial communities and soil chemical features associated with commercial production of the medicinal mushroom Ganoderma lingzhi in soil
Source: Sci Rep. 2019 Nov 1;9:15839. doi: 10.1038/s41598-019-52368-2 (PMC6825212; doi:10.1038/s41598-019-52368-2)
Supplement: Supplementary file 1 — Supplementary table [file 41598_2019_52368_MOESM1_ESM.docx]

Microbial communities and soil chemical features associated with commercial production of the medicinal mushroom *Ganoderma lingzhi* in soil

Le-Qin Ke ^1,^*, Pu-Dong Li ^2^, Jian-Ping Xu ^3^, Qiu-Shuang Wang ^4^, Liang-Liang Wang ^1^ and Hui-Ping Wen ^1^

^1^ College of Ecology, Lishui University, Lishui City, Zhejiang Province, 323000, P. R. China

^2^ State Key Laboratory of Rice Biology, Institute of Biotechnology, Zhejiang University, Hangzhou, 310058, China

^3^ Department of Biology, McMaster University, 1280 Main St. West, Hamilton Ontario, L8S 4K1, Canada

^4^ Tea Research Institute, Guangdong Academy of Agricultural Sciences, Guangzhou, Guangdong 510006, P. R. China

***** Correspondence: 63225706@qq.com; Tel: +86 (0)5782271308

**Table S1** The sequences of the primers and barcodes.

| **Sample ID** | **Barcode Sequence** | **Linker Primer Sequence** |  |  |  |
| --- | --- | --- | --- | --- | --- |
| **Primer and barcode sequences for the 16S region** | | | | | |
| RS4.2 | GGTAGC,TCAAGT | GTGCCAGCMGCCGCGGTAA,GGACTACHVGGGTWTCTAAT | | | |
| RS1.1 | GGTAGC,AGTCAA | GTGCCAGCMGCCGCGGTAA,GGACTACHVGGGTWTCTAAT | | | |
| RS1.2 | GGTAGC,AGTTCC | GTGCCAGCMGCCGCGGTAA,GGACTACHVGGGTWTCTAAT | | | |
| RS1.3 | GGTAGC,ATGTCA | GTGCCAGCMGCCGCGGTAA,GGACTACHVGGGTWTCTAAT | | | |
| RS2.1 | GGTAGC,CCGTCC | GTGCCAGCMGCCGCGGTAA,GGACTACHVGGGTWTCTAAT | | | |
| RS2.2 | GGTAGC,GTAGAG | GTGCCAGCMGCCGCGGTAA,GGACTACHVGGGTWTCTAAT | | | |
| RS2.3 | GGTAGC,GTCCGC | GTGCCAGCMGCCGCGGTAA,GGACTACHVGGGTWTCTAAT | | | |
| RS3.1 | GGTAGC,GTGAAA | GTGCCAGCMGCCGCGGTAA,GGACTACHVGGGTWTCTAAT | | | |
| RS3.2 | GGTAGC,GTGGCC | GTGCCAGCMGCCGCGGTAA,GGACTACHVGGGTWTCTAAT | | | |
| RS3.3 | GGTAGC,GGCCTG | GTGCCAGCMGCCGCGGTAA,GGACTACHVGGGTWTCTAAT | | | |
| RS4.1 | GGTAGC,TTACTG | GTGCCAGCMGCCGCGGTAA,GGACTACHVGGGTWTCTAAT | | | |
| RS4.3 | GGTAGC,AGCAGT | GTGCCAGCMGCCGCGGTAA,GGACTACHVGGGTWTCTAAT | | | |
| **Primer and barcode sequences for the ITS region** | | | | | |
| RS1.1 | GCCAAT,GGCTAC | TCCGTAGGTGAACCTGCGG,GCTGCGTTCTTCATCGATGC | | | |
| RS1.2 | GCCAAT,CTTGTA | TCCGTAGGTGAACCTGCGG,GCTGCGTTCTTCATCGATGC | | | |
| RS1.3 | GCCAAT,AGTCAA | TCCGTAGGTGAACCTGCGG,GCTGCGTTCTTCATCGATGC | | | |
| RS2.1 | GCCAAT,AGTTCC | TCCGTAGGTGAACCTGCGG,GCTGCGTTCTTCATCGATGC | | | |
| RS2.2 | GCCAAT,ATGTCA | TCCGTAGGTGAACCTGCGG,GCTGCGTTCTTCATCGATGC | | | |
| RS2.3 | GCCAAT,CCGTCC | TCCGTAGGTGAACCTGCGG,GCTGCGTTCTTCATCGATGC | | | |
| RS3.1 | GCCAAT,GTAGAG | TCCGTAGGTGAACCTGCGG,GCTGCGTTCTTCATCGATGC | | | |
| RS3.2 | GCCAAT,GTCCGC | TCCGTAGGTGAACCTGCGG,GCTGCGTTCTTCATCGATGC | | | |
| RS3.3 | GCCAAT,GTGAAA | TCCGTAGGTGAACCTGCGG,GCTGCGTTCTTCATCGATGC | | | |
| RS4.1 | GCCAAT,GTGGCC | TCCGTAGGTGAACCTGCGG,GCTGCGTTCTTCATCGATGC | | | |
| RS4.2 | GCCAAT,GTTTCG | TCCGTAGGTGAACCTGCGG,GCTGCGTTCTTCATCGATGC | | | |
| RS4.3 | CGATGT,GGCTAC | TCCGTAGGTGAACCTGCGG,GCTGCGTTCTTCATCGATGC | | | |

**Table S2** Analysis of 16S rRNA sequencing reads.

| Sample name | Raw reads | Quality reads | Q20 | Q30 | GC % | Avg. length  (bp) | Total OTUs |
| --- | --- | --- | --- | --- | --- | --- | --- |
| RS1.1 | 61,885 | 59,878 | 98.91 | 97.97 | 56.46 | 253 | 5236 |
| RS1.2 | 67,038 | 64,829 | 98.99 | 98.08 | 56.64 | 253 | 5115 |
| RS1.3 | 76,576 | 73,903 | 98.95 | 98 | 56.55 | 253 | 5847 |
| RS2.1 | 50,820 | 49,035 | 98.9 | 97.95 | 56.51 | 253 | 4926 |
| RS2.2 | 76,178 | 73,680 | 98.89 | 97.94 | 56.75 | 253 | 5569 |
| RS2.3 | 76,528 | 74,073 | 99.06 | 98.2 | 56.52 | 253 | 4233 |
| RS3.1 | 47,987 | 46,355 | 98.88 | 97.93 | 56.88 | 253 | 4272 |
| RS3.2 | 55,022 | 53,201 | 98.88 | 97.92 | 56.81 | 253 | 5012 |
| RS3.3 | 58,990 | 54,197 | 98.64 | 97.41 | 56.88 | 262 | 4825 |
| RS4.1 | 35,729 | 34,466 | 98.81 | 97.77 | 57.07 | 253 | 3666 |
| RS4.3 | 61,637 | 59,498 | 98.85 | 97.88 | 56.76 | 253 | 3938 |
| RS4.2 | 64,774 | 61,370 | 98.76 | 97.75 | 56.84 | 253 | 4517 |

**Table S3** Analysis of sequencing reads for the ITS region.

| Sample Name | Raw reads | Quality reads | Avg. length (bp) | Q20 | Q30 | GC % | Total OTUs |
| --- | --- | --- | --- | --- | --- | --- | --- |
| RS1.1 | 60,179 | 49,468 | 238 | 99.12 | 98.3 | 48.91 | 2061 |
| RS1.2 | 60,089 | 47,036 | 247 | 99.07 | 98.21 | 46.33 | 1883 |
| RS1.3 | 79,480 | 35,160 | 314 | 98.41 | 97.23 | 43.07 | 726 |
| RS2.1 | 33,816 | 30,520 | 226 | 99.09 | 98.28 | 43.94 | 1957 |
| RS2.2 | 70,784 | 65,460 | 240 | 99.13 | 98.32 | 47.54 | 1391 |
| RS2.3 | 37,563 | 21,832 | 268 | 98.94 | 97.99 | 45.19 | 855 |
| RS3.1 | 30,492 | 25,982 | 231 | 99.09 | 98.29 | 43.44 | 1550 |
| RS3.2 | 62,039 | 56,724 | 242 | 99.24 | 98.48 | 47.47 | 1068 |
| RS3.3 | 70,674 | 63,461 | 236 | 99.14 | 98.35 | 49.22 | 1138 |
| RS4.1 | 67,136 | 44,038 | 252 | 98.97 | 98.03 | 47.58 | 1489 |
| RS4.2 | 77,606 | 72,807 | 237 | 99.1 | 98.21 | 44.43 | 1155 |
| RS4.3 | 58,007 | 31,224 | 250 | 98.77 | 97.7 | 46 | 1082 |

**Table S4** The 16S reads that were classified into different bacterial taxonomy levels.

| Sample name | Kingdom | Phylum | Class | Order | Family | Genus | Species |
| --- | --- | --- | --- | --- | --- | --- | --- |
| RS1.1 | 53748 | 53083 | 51850 | 45628 | 31503 | 12198 | 2135 |
| RS1.2 | 59664 | 58784 | 57667 | 51141 | 34902 | 13634 | 2239 |
| RS1.3 | 67159 | 66378 | 64943 | 57655 | 39408 | 16205 | 2239 |
| RS2.1 | 44335 | 43690 | 42759 | 37471 | 25165 | 9670 | 2640 |
| RS2.2 | 67103 | 66100 | 64742 | 56469 | 39386 | 15308 | 4406 |
| RS2.3 | 66830 | 65852 | 64450 | 56413 | 37754 | 15710 | 4112 |
| RS3.1 | 42572 | 41791 | 40874 | 35861 | 24392 | 7094 | 1459 |
| RS3.2 | 47761 | 47109 | 46141 | 40097 | 26080 | 9571 | 1846 |
| RS3.3 | 48530 | 46666 | 45642 | 40477 | 27673 | 9214 | 2798 |
| RS4.1 | 31858 | 31365 | 30821 | 27352 | 19236 | 5869 | 960 |
| RS4.2 | 55495 | 54928 | 54228 | 46130 | 33310 | 10444 | 1973 |
| RS4.3 | 54425 | 53551 | 52781 | 45176 | 31653 | 8888 | 1230 |

**Table S5** ITS reads that were classified into different bacterial taxonomy levels.

| Sample name | Kingdom | Phylum | Class | Order | Family | Genus | Species |
| --- | --- | --- | --- | --- | --- | --- | --- |
| RS1.1 | 45387 | 39924 | 39872 | 39871 | 39807 | 39550 | 39550 |
| RS1.2 | 43449 | 37327 | 37253 | 37252 | 37126 | 36579 | 36570 |
| RS1.3 | 33396 | 31044 | 31033 | 31033 | 30978 | 30940 | 30940 |
| RS2.1 | 27482 | 23212 | 22979 | 22979 | 22884 | 22037 | 22037 |
| RS2.2 | 63302 | 61206 | 61108 | 61105 | 60817 | 58160 | 58160 |
| RS2.3 | 20084 | 18075 | 18058 | 18058 | 18046 | 17876 | 17876 |
| RS3.1 | 23195 | 18619 | 18510 | 18509 | 18370 | 17892 | 17892 |
| RS3.2 | 55636 | 52253 | 52229 | 52225 | 52170 | 52121 | 52121 |
| RS3.3 | 61755 | 59167 | 59094 | 59079 | 59047 | 58749 | 58749 |
| RS4.1 | 40869 | 31551 | 31460 | 31449 | 31339 | 30989 | 30988 |
| RS4.2 | 71071 | 67031 | 66996 | 66996 | 66923 | 66685 | 66685 |
| RS4.3 | 27235 | 22505 | 22415 | 22405 | 22366 | 22317 | 22317 |
